# Murraya *koenigii* leaf extract inhibits proteasome activity and induces cell death in breast cancer cells

**DOI:** 10.1186/1472-6882-13-7

**Published:** 2013-01-09

**Authors:** Bindu Noolu, Rajanna Ajumeera, Anitha Chauhan, Balakrishna Nagalla, Raghunath Manchala, Ayesha Ismail

**Affiliations:** 1 Department of Endocrinology & Metabolism, National Institute of Nutrition, Hyderabad, India; 2 Department of Stem Cell Research, National Institute of Nutrition, Hyderabad, India; 3 Department of Statistics, National Institute of Nutrition, Hyderabad, India

**Keywords:** Murraya *koenigii*, 26S proteasome, Breast cancer, Polyphenols, Methanolic extract, Proteasome inhibitor

## Abstract

**Background:**

Inhibition of the proteolytic activity of 26S proteasome, the protein-degrading machine, is now considered a novel and promising approach for cancer therapy. Interestingly, proteasome inhibitors have been demonstrated to selectively kill cancer cells and also enhance the sensitivity of tumor cells to chemotherapeutic agents. Recently, polyphenols/flavonoids have been reported to inhibit proteasome activity. Murraya *koenigii* Spreng, a medicinally important herb of Indian origin, has been used for centuries in the Ayurvedic system of medicine. Here we show that Murraya *koenigii* leaves (curry leaves), a rich source of polyphenols, inhibit the proteolytic activity of the cancer cell proteasome, and cause cell death.

**Methods:**

Hydro-methanolic extract of curry leaves (CLE) was prepared and its total phenolic content [TPC] determined by, the Folin-Ciocalteau’s method. Two human breast carcinoma cell lines: MCF-7 and MDA-MB-231 and a normal human lung fibroblast cell line, WI-38 were used for the studies. Cytotoxicity of the CLE was assessed by the MTT assay. We studied the effect of CLE on growth kinetics using colony formation assay. Growth arrest was assessed by cell cycle analysis and apoptosis by Annexin-V binding using flow cytometry. Inhibition of the endogenous 26S proteasome was studied in intact cells and cell extracts using substrates specific to 20S proteasomal enzymes.

**Results:**

CLE decreased cell viability and altered the growth kinetics in both the breast cancer cell lines in a dose-dependent manner. It showed a significant arrest of cells in the S phase albeit in cancer cells only. Annexin V binding data suggests that cell death was via the apoptotic pathway in both the cancer cell lines. CLE treatment significantly decreased the activity of the 26S proteasome in the cancer but not normal cells.

**Conclusions:**

Our study suggests M. *koenigii* leaves to be a potent source of proteasome inhibitors that lead to cancer cell death. Therefore, identification of active component(s) from the leaf extract could lead to the development of anti-cancer agents which could be useful in the treatment of different types of cancers.

## Background

Breast cancer is the second most prevalent cancer in the world next only to lung cancer [1] and is a major public health problem in developing countries like India. Every year 75,000 new cases of breast cancer are reported in India. The increase in the number of cases has been attributed to factors such as genetics, environmental pollution, urbanization and changing food habits.

Murraya *koenigii* Spreng (curry-leaf tree), is a small aromatic tree belonging to the family Rutaceae. It is a tropical to sub-tropical tree native to India. Of the 14 global species belonging to the genus Murraya, only two are available in India, namely, M. *koenigii* and M. *paniculata*. Of the two M. *koenigii* is more popular due to its large spectrum of medicinal properties. M. *koenigii* leaves have a slightly pungent, bitter and feebly acidulous taste and these characteristics are retained even after drying. Fresh and dried curry leaves are extensively used in South Indian culinary practices for seasoning and flavouring dishes [2]. Different parts of the plant such as leaves, root, bark and fruit are known to possess various biological activities. Traditionally, this plant is used in Indian systems of medicine for a variety of ailments and also used as a tonic, stomachic, and carminative [3-5].

The major chemical constituents of the plant reported are carbazole alkaloids, coumarins and flavonoids [6]. M. *koenigii* leaf extracts exhibit hypoglycemic and hypolipidemic effects in experimental animals [7-9]. Carbazole alkaloids and methanolic extracts of M. *koenigii* are also reported to possess anti-oxidative [10-12], anti-diarrheal and anti-trichomonal activities [13,14]. M. *koenigii* leaf extracts reduced blood cholesterol and glucose levels in ob/ob mice [15]. Methanolic extract of M. *koenigii* leaves possess anti-inflammatory [16] and immunomodulatory activity [17]. Mahanine, a carbazole alkaloid purified from M. *koenigii* leaves has apoptotic effects in human leukemia cells [18-20].

We and others have shown that a hydro-methanolic extract of M. *koenigii* leaves is rich in phenolic content [10,21]. Polyphenols have a wide spectrum of biological activities, including anti-oxidant, anti-inflammatory and metal-chelating properties [22,23]. Recent studies have shown that naturally occurring polyphenols/flavanoids modulate the functionality of the 26S proteasome, a multi-enzymatic, multi-catalytic complex localized both in the cytoplasm and nucleus of eukaryotic cells [24,25]. The 26S proteasome is a huge 2.4 MDa complex comprising of two sub-complexes – the 19S regulatory subunit and the 20S catalytic subunit [26]. The 20S sub-unit possesses at least three distinct activities, which are associated with the three different β subunits respectively: chymotrypsin-like activity (β5), trypsin-like activity (β2) and the caspase-like activity (β1) [27]. The 26S proteasome is the major non-lysosomal pathway of protein degradation in eukaryotic cells. This proteolytic machine is involved in the degradation of oxidized, unfolded and misfolded proteins and antigen presentation [28-31]. It regulates several cellular processes such as apoptosis, signal transduction, cell-cycle regulation and cell differentiation [32]. Two important functions of the proteasome system are to promote tumor cell proliferation and protect tumor cells against apoptosis [27,33,34].

In the present work, we demonstrate for the *first time* that the hydro-methanolic extract of M. *koenigii* leaves rich in phenolic content, potently inhibits the activity of the proteasome both *in vitro* and *in vivo*. The CLE induced cell death in two breast cancer cell lines in a time and dose-dependent manner. The leaf extract altered the growth kinetics of the cancer cells in a dose-dependent manner as demonstrated by the colony formation assay. Cancer cells but not normal cells were arrested in the S phase of the cell cycle. Annexin V binding experiments demonstrate that apoptosis was induced by CLE in both the breast carcinoma cell lines.

## Methods

### Chemicals & reagents

Dulbecco’s Modified Eagle’s Medium (DMEM)- cell culture media, antibiotic-antimycotic mix, sodium pyruvate, non-essential amino acid mix and stable glutamine were purchased from Himedia (Mumbai, India); fetal bovine serum (FBS) was purchased from (GIBCO, Invitro-gen USA), 3-[4, 5-dimethyltiazol-2-yl]-2.5-diphenyl-tetrazolium bromide (MTT), Dimethylsulfoxide (DMSO), Propidium Iodide, Ribonuclease A, Dithiothreitol (DTT), 3-[(3-Cholamidopropyl)dimethylammonio]-1-propanesulfonate (CHAPS), Ethylene diamine tetra acetic acid (EDTA), Phenyl methyl sulfoxide (PMSF), Crystal-violet, Sodium dodecyl sulphate (SDS) and 4-(2-Hydroxyethyl)piperazine-1-ethanesulfonic acid N-(2-Hydroxyethyl)piperazine-N′-(2-ethanesulfonic acid) **(**HEPES) were purchased from Sigma-Aldrich (St Louis, MO, USA). The fluorogenic proteasomal peptide substrates Suc-LLVY-AMC (chymotrypsin-like substrate), BOC-Leu-Arg-Arg-AMC (trypsin -like substrate) and Z-Leu-Leu-Glu-AMC (caspase- like substrate) and MG-132 (carbobenzoxy-Leu-Leu-leucinal - a specific inhibitor of the 26S proteasome) were procured from ENZO Life sciences, USA. 20S rabbit proteasome was purchased from Boston Biochem, USA. All other reagents were procured from Qualigens fine chemicals (Mumbai, India). Annexin staining was done using a kit (Annexin V-FITC Apoptosis detection kit; BD Pharmingen, San Jose, CA, USA).

### Preparation of curry leaf extracts

Curry leaves were collected from the local area from a single tree. Identity of the curry leaves was confirmed by Dr. B. Pratibha Devi, Professor and Head, Department of Botany, Osmania University, Hyderabad, India. A voucher specimen (voucher no: 068) was deposited in a herbarium at the Department of Botany, Osmania University, Hyderabad, India. The leaves were washed and air dried in shade for 3 weeks. After drying, the leaves were ground to a fine powder using an electric mixer grinder. The leaf powder was extracted with 80% methanol in water by keeping on a vortex mixer for 3-4days. This was followed by centrifugation of the extract at 5000 rpm for 30 min. The supernatant was filtered using a 0.4 μm filter (Millipore). The resultant Methanol:Water extract was stored at −20°C and was used for all our studies. These extracts designated as ‘**CLE**’, were used in the cell-culture assays at different doses based on their total phenolic content [equivalent to μg of gallic acid (GAE)] measured spectrophotometrically by the Folin-Ciocalteau method.

### Total Phenolic Content (TPC)

The total phenolic content of the extract was determined with Folin–Ciocalteau’s reagent using Gallic acid as a standard [35]. Different concentrations of Gallic acid standards (20-100 μg/μl) and CLE samples were taken in glass test tubes and volume was made up to 150 μl with distilled water. 750 μl of 10% Folin’s reagent was added and kept for 5 minutes at room temperature, followed by addition of 750 μl of 6% Na_2_CO_3_ and vortexed for 5 minutes. The tubes were then incubated for 90 minutes at room temperature. The absorbance was measured at 725 nm in a Hitachi double beam spectrophotometer. The final concentration of the total polyphenols present in the extract was expressed as μg of Gallic Acid Equivalents (GAEs).

### Cell lines

MCF-7 and MDA-MB-231 (breast carcinoma cell lines) and WI-38 (normal human lung fibroblasts) were obtained from the National Centre for Cell Sciences, Pune, India. Cells were maintained in DMEM supplemented with 10% FBS, 2 mM Higluta-XL, 100 units/ml penicillin, 100 μg/ml streptomycin and 0.5 ng/ml amphotericin B, 1 mM sodium pyruvate and 1X non-essential amino acid mixture. Cells were maintained and grown in a humidified atmosphere at 37°C and 5% CO_2_. WI-38 cells were grown for no more than 30 passages, as recommended by European Collection of Cell Cultures (ECACC).

### Cell viability/proliferation assay

Cell viability was determined by quantification of 3-(4,5-dimethylthiazol-2-yl)-2,5-di-phenyltetrazolium bromide (MTT) reduction by mitochondrial dehydrogenases. In brief, 1 × 10^5^ cells/well were plated in a 96-well plate and incubated with different concentrations of the CLE for either 12 h or 24 h or with MG-132 for 24 h. Following this, MTT was added to a final concentration of 100 μg/well and further incubated for 3 h at 37°C. The formazan dye crystals formed were solubilized in DMSO and the plate was incubated at room temperature for 1 h. The absorbance was measured at 595 nm in an ELISA microplate reader (Biotek, New York, USA). All samples were assayed in triplicate in three independent experiments. Absorbance values plotted are the mean from three independent experiments and the results are expressed as percentage of the control, which was considered to be 100%.

### Colony formation assay

MCF-7 and MDA-MB-231 cells were plated in duplicate at a density of 1 × 10^4^ and 1 × 10^3^ cells/ml respectively in 6-well plates. Next day, the cells were treated with varying concentrations of CLE. Plates were incubated at 37°C and 5% CO_2_ for one week. After a week, the colonies were fixed with 4% formaldehyde for 15mins followed by staining with 0.005% crystal violet. The colonies were photographed with a digital Nikon D90 camera. Three independent experiments were done with each cell line.

### Cell cycle analysis and annexin V binding assay

Cell cycle analysis: MCF-7 or MDA-MB-231 or WI-38 cells were plated at a density of 1.5 × 10^6^ cells in a 10 cm dish. Next day, cells were treated with different doses of the CLE. After 24 h incubation, cells were harvested and suspended in 1X-PBS containing 2% FBS. The cells were fixed with 70% ethanol at 4°C for 1 h followed by the addition of propidium iodide (5 μg/ μl) and RNase (10 μg/ μl) and further incubated for 3 h at 4°C. The DNA content was evaluated in a flow cytometer (BD FACS ARIAII, USA). The data was analyzed using Modfit software (BD Biosciences, USA).

Annexin-V staining: MCF-7 or MDA-MB-231 cells were plated at a density of 1.5 × 10^6^ cells in a 10 cm dish. Next day, cells were treated with different doses of the CLE. After 24 h incubation, cells were washed with 1X-PBS and re-suspended in 100μl binding buffer (supplied by the vendor). Cells were stained with Annexin V-FITC and propidium iodide according to the manufacturer’s protocol before analysis by flow cytometry.

### Inhibition of purified 20S proteasome activity

Chymotrypsin-like activity of the purified 20S proteasome was measured as follows: In brief, 200 ng of purified 20S proteasome was incubated in 200μl of assay buffer (50 mmol/L Tris–HCl, pH 8.0 containing 0.035% SDS) with or without different concentrations of CLE and 40 μM substrate Suc-Leu-Leu-Val-Tyr-AMC (for chymotrypsin-like activity) and incubated for 2h at 37°C. The free 7-amino-4-methylcoumarin (AMC) liberated was measured fluorimetrically using a multi-mode reader [Spectra Max M5] using an excitation filter (380 nm) and emission filter (460 nm). The data is plotted as mean (+/− standard error) and is expressed as a percentage of the control, which was considered to be 100%. The assay was repeated thrice.

### Inhibition of 26S proteasome activity in intact cells

To measure inhibition of the proteasome activity in living tumor cells, MCF-7 or MDA-MB-231 or WI-38 cells were plated at a density of 1 × 10^4^ in a 24 well plate. Next day, cells were treated with or without the CLE at the indicated concentrations. After 24 h of treatment, the media was aspirated out and 500 μl of 1X-PBS was added followed by addition of fluorogenic substrates (20 μM final concentration) specific for the chymotrypsin-like (Ch-L), trypsin-like (T-L) and caspase-like (Cp-L) activities of the 20S proteasome. The plate was then incubated for 2 h at 37°C. 200 μl of the 1X- PBS was then transferred into a black plate and the free 7-amino-4-methylcoumarin (AMC) liberated was measured fluorimetrically in a multi-mode reader [Spectra Max M5] at excitation (380 nm) and emission (460 nm). The results are displayed as mean (+/− standard error) and are expressed as a percentage of the control, which was considered to be 100%. All samples were assayed in triplicate in three independent experiments.

### Inhibition of 26S proteasome activity in cell extracts

MCF-7 or MDA-MB-231 cells (1 × 10^7^) were harvested, washed twice in 1X-PBS and re-suspended in 1 ml ATP/DTT lysis buffer [10 mmol/L Tris–HCl (pH 7.8), 5 mmol/L ATP, 0.5 mmol/l DTT, 5 mmol/L MgCl_2_]. Cells were incubated on ice for 10 minutes, followed by sonication for 15 seconds. The lysate was centrifuged at 2000 rpm for 10 minutes at 4°C. The supernatant enriched in 26S proteasomes is the cell extract which was used for the assay. This was mixed with glycerol (20% final concentration), aliquoted and stored at −80°C, and was stable for at least 1 month. The total protein content of the cell extract was estimated by the Bicinchoninic Acid (BCA) method using a kit (Bangalore Genei, Bangalore, India). The assay was carried out in a total of 200 μl reaction volume containing proteasome extract (50 μg protein), 50 mM EDTA, varying concentrations of the CLE/MG-132 and 50 μM of the proteasomal fluorogenic substrates and incubated for 2 h at 37°C. The amount of free 7-amino-4-methylcoumarin (AMC) liberated was measured fluorimetrically. The results are expressed as mean (+/− standard error) as a percentage of the control, which was considered to be 100%. All samples were assayed in triplicate in three independent experiments.

### Statistical analysis

All experiments were performed in triplicates and repeated at least three times and the data are presented as mean+/− SEM. Mean values were compared across concentrations of CLE using non-parametric test of Kruskal-Wallis one-way ANOVA for each cell line using the SPSS statistical software. Differences between groups were considered significant at α (probability) level of </= 0.05.

## Results

### M. *koenigii* leaf extract alters viability and growth kinetics of breast cancer cells

**Figure 1:**
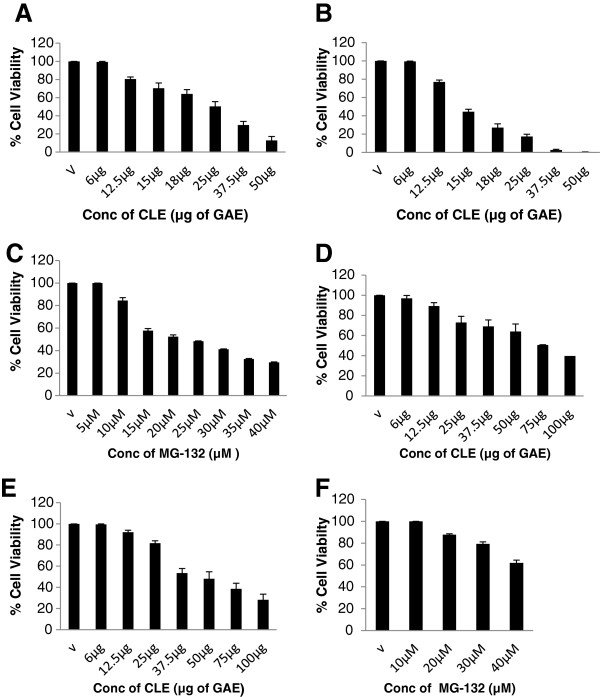
**Curry leaf extract decreases cell viability of breast cancer cells:** Panels **A** &**B** shows results of MTT assay after 12 h and 24 h treatment with CLE, whereas Panel **C** shows results of MTT assay after 24 h treatment with MG-132, a specific proteasome inhibitor respectively in **MDA-MB-231 cell line**. Panels **D** &**E** shows results of MTT assay after 12 h and 24 h treatment with CLE, whereas Panel **F** shows results of MTT assay after 24 h treatment with MG-132, in **MCF-7 cell line**. The data represents mean+/− SEM of three independent experiments.

The TPC of the methanolic extract of curry leaves was 3 μg of GAEs/μl of the CLE. MTT assays were performed with different concentrations of CLE (GAE) in both the cell lines at the 12 h and 24 h time points to assess the effect of the extract on cell viability. There was a significant (p<0.05) time and dose-dependent decrease in cell viability in both the cell lines. As expected, the decrease in cell viability observed after 24 h of treatment was higher compared to 12 h. In addition, MDA-MB-231 cells (Figure 1A and 1B) appeared to be more sensitive to CLE induced cell death than MCF-7 cells (Figure 1D and 1E). 24 h post treatment, a 50% reduction in cell viability was observed in MDA-MB-231 and MCF-7 cells at 15 μg and 37.5 μg GAE of CLE respectively. As a positive control, the effect of MG-132- a specific inhibitor of the proteolytic activity of the 26S proteasome was also assessed on cell viability in both the breast cancer cell lines. 24 h post treatment with MG-132, a 50% reduction in cell viability was observed at 20 μM and >40 μM of MG-132 in MDA-MB-231and MCF-7 cells respectively (Figure 1C and 1F).

**Figure 2:**
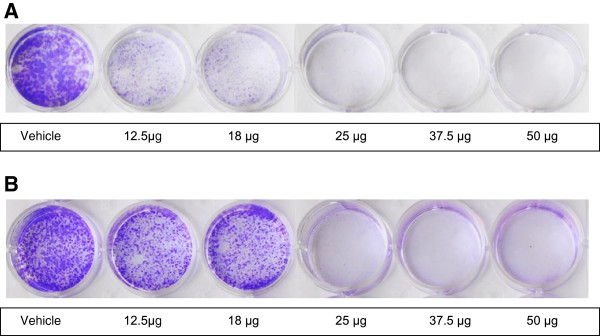
**Curry leaf extract alters growth kinetics of breast cancer cells:** Panel **A** depicts results of colony formation assay in **MDA-MB-231 cell line**. Panel **B** depicts results of colony formation assay in **MCF-7 cell line**. Cells were grown in 6-well plates and treated with various concentrations of the CLE (0- 50 μg GAE). After a week cells were stained with crystal violet and photographed.

To test the effect of CLE on growth kinetics, MCF-7 and MDA-MB-231 cells were seeded at a lower density and treated with different concentrations of the CLE. After incubation for a week, it was observed that at a dose of 25 μg GAE of CLE, no colonies were found in either MDA-MB-231 (Figure 2A) or MCF-7 cells (Figure 2B). In line with our observations on cell viability (MTT assay), MDA-MB-231 cells appeared to be more sensitive than MCF-7 cells. This is supported by our findings where a lesser concentration of CLE was needed to inhibit the formation of colonies in MDA-MB-231 cells in comparison to MCF-7 cells.

### M. *koenigii* leaf extract induces growth arrest and apoptosis in breast cancer cells

**Figure 3:**
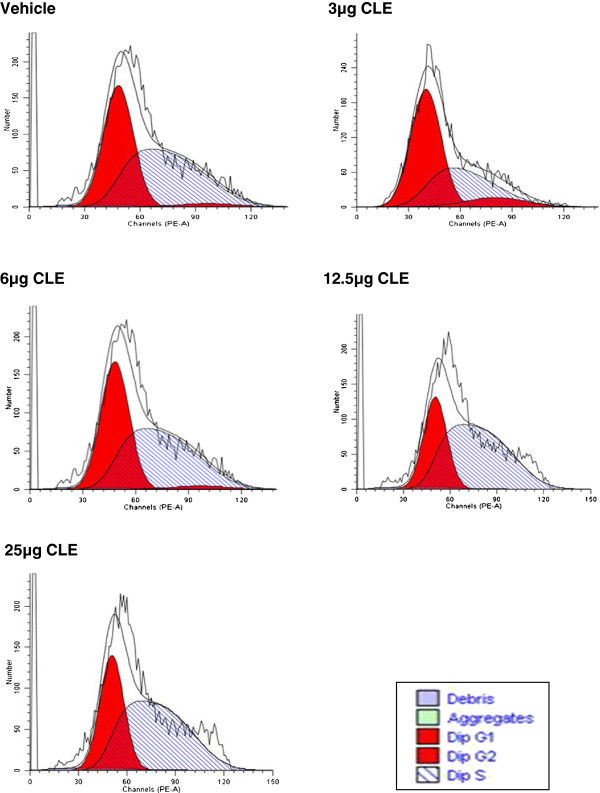
**Cell cycle arrest by CLE in the MDA-MB-231 breast carcinoma cell line.** Cell cycle analysis of MDA-MB-231 cells treated with varying concentrations of CLE for 24 h was done by flow cytometry.

**Figure 4:**
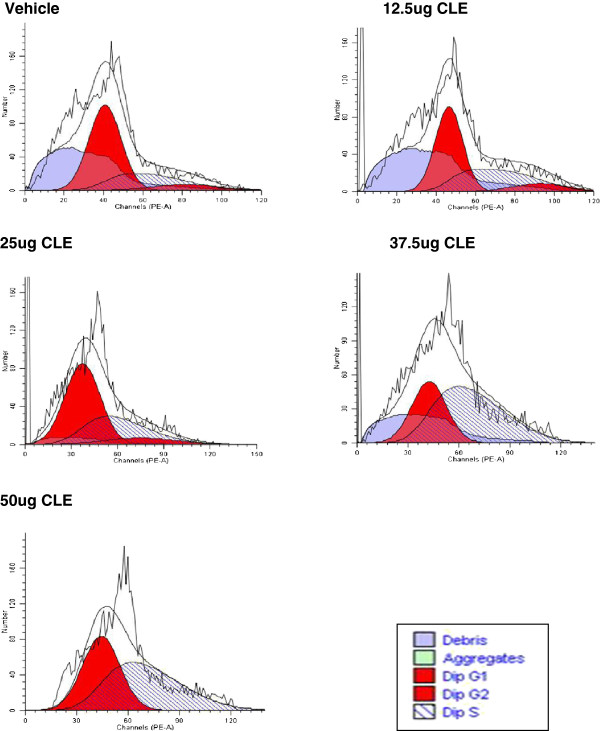
**Cell cycle arrest by CLE in the MCF-7 breast carcinoma cell line.** Cell cycle analysis of MCF-7 cells treated with varying concentrations of CLE for 24 h was done by flow cytometry.

**Figure 5:**
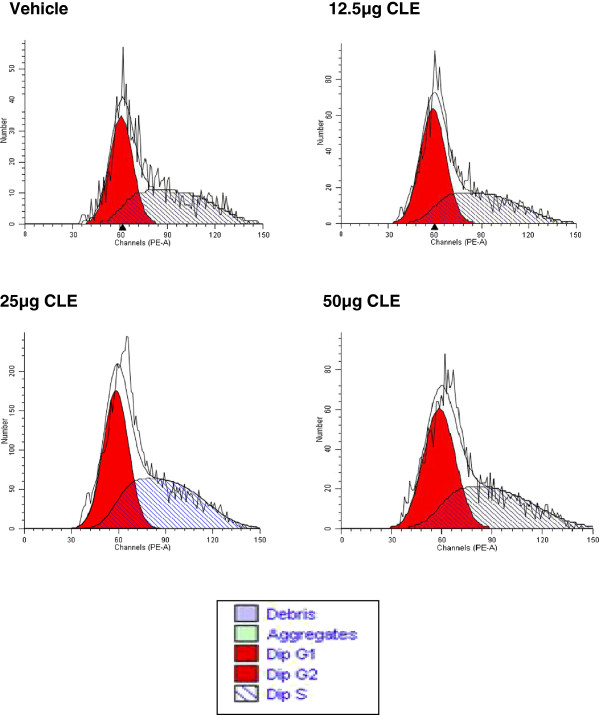
**CLE treatment does not arrest cell cycle in the WI-38 normal lung fibroblast cell line.** Cell cycle analysis of WI-38 cells treated with varying concentrations of CLE for 24 h was done by flow cytometry.

Cell cycle experiments were done to determine whether CLE treatment arrested growth in MDA-MB-231 and MCF-7 cells. In both the breast carcinoma cell lines CLE treatment showed a dose-dependent arrest in the S phase of the cell cycle resulting in complete inhibition of cell proliferation (Figures 3 and 4; Tables 1 and 2). That inhibition of cell proliferation denoted by G2-M phase was observed at 12.5 μg GAE in MDA-MB-231 cells, whereas, in MCF-7 cells it was seen at 37.5 μg GAE, corroborates the greater sensitivity of MDA-MB-231 than MCF-7 cells to the CLE induced effects. Interestingly, CLE had no effect on cell cycle in the normal WI-38 cell line at any of the concentrations tested (Figure 5 and Table 3), indicating that CLE could arrest growth only in cancer but not normal cells.

**Table 1:** Distribution [%] of MDA-MB-231 cells in different phases of the cell cycle

**CELL PHASE**	**VEHICLE**	**3 μg**	**6 μg**	**12.5 μg**	**25 μg**
**G0-G1**	**43.98**	**54.15**	**44.53**	**33.9**	**37.3**
**S**	**52.07**	**37.26**	**53**	**66.10**	**62.6**
**G2-M**	**3.95**	**8.59**	**2.48**	**0.0**	**0.0**

**Table 2:** Distribution [%] of MCF-7 cells in different phases of the cell cycle

**CELL PHASE**	**VEHICLE**	**12.5 μg**	**25 μg**	**37.5 μg**	**50 μg**
**G0-G1**	**63.51**	**52.24**	**56.42**	**35.62**	**44.46**
**S**	**27.89**	**38.22**	**34.68**	**64.38**	**55.54**
**G2-M**	**8.60**	**9.59**	**8.89**	**0.0**	**0.0**

**Table 3:** Distribution [%] of WI-38 cells in different phases of the cell cycle

**CELL PHASE**	**VEHICLE**	**12.5 μg**	**25 μg**	**50 μg**
**G0-G1**	**48.7**	**55.83**	**48.57**	**52.8**
**S**	**50.35**	**44.16**	**51.43**	**47.20**
**G2-M**	**0.87**	**0.00**	**0.00**	**0.00**

**Figure 6:**
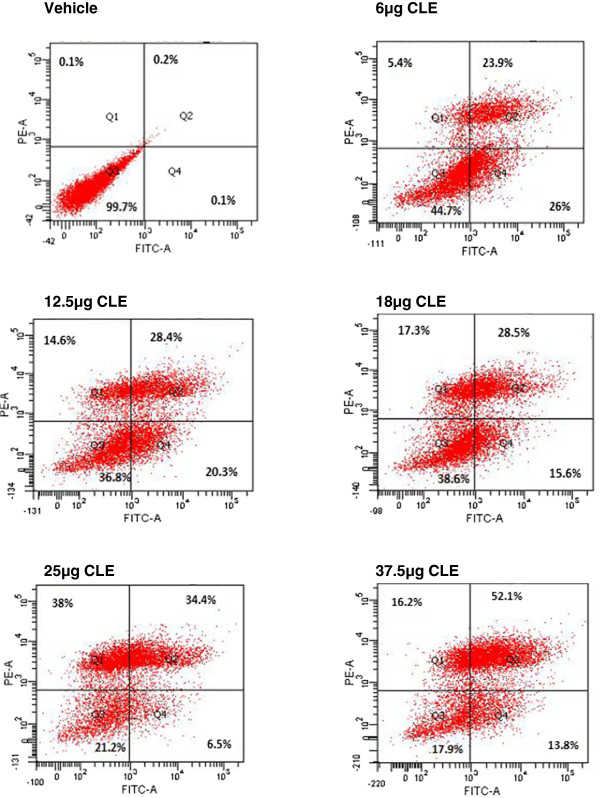
**Exposure to CLE induces apoptosis in the MDA-MB-231 breast carcinoma cell line.** Phosphatidylserine levels were detected by annexin V-FITC binding. MDA-MB-231 cells were treated with varying concentrations of the CLE for 24 h and stained with FITC-conjugated annexin V and propidium iodide (PI).

**Figure 7:**
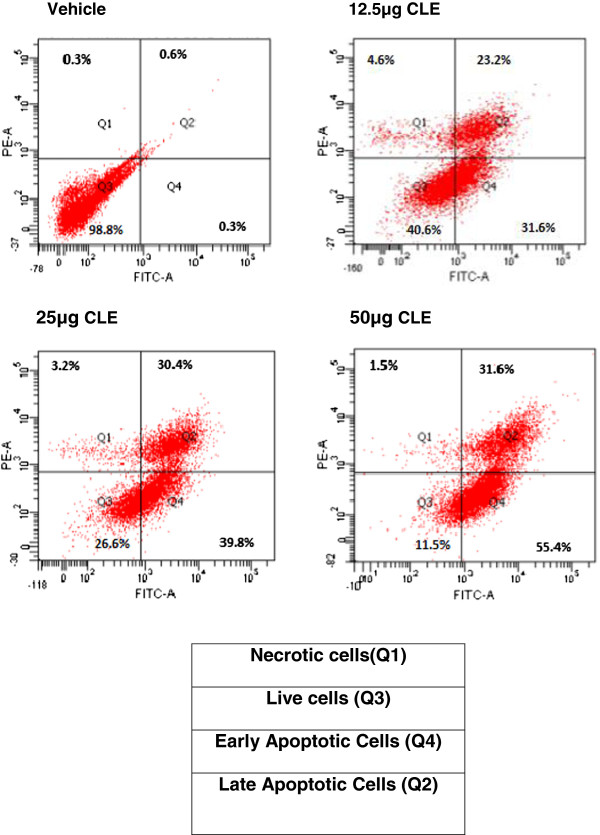
**Exposure to CLE induces apoptosis in the MCF-7 breast carcinoma cell line.** Phosphatidylserine levels were detected by annexin V-FITC binding. MCF-7 cells were treated with varying concentrations of the CLE for 24 h and stained with FITC-conjugated annexin V and propidium iodide (PI).

Further, Annexin-V binding experiments were conducted in both cell lines to determine the probable mechanism of cell death. That 6μg CLE resulted in 45% of live MDA-MB-231 cells whereas, a dose of 12.5 μg CLE was required for a similar effect in MCF-7 cells not only confirms the greater sensitivity of MDA-MB-231 than MCF-7 cells but also suggests apoptosis to be the probable mechanism of cell death. This is confirmed by our finding that CLE demonstrated a dose dependent increase in the % of apoptotic cells in both MDA-MB-231 and MCF-7 cells. 50% of cells were apoptotic with 6 μg CLE in MDA-MB-231 cell line and it increased to 66% at a dose of 37.5 μg CLE (Figure 6). A similar dose dependent increase in the % apoptotic cells was seen in MCF-7 cells (Figure 7).

### M. *koenigii* leaf extract inhibits 20S purified proteasome activity

**Figure 8:**
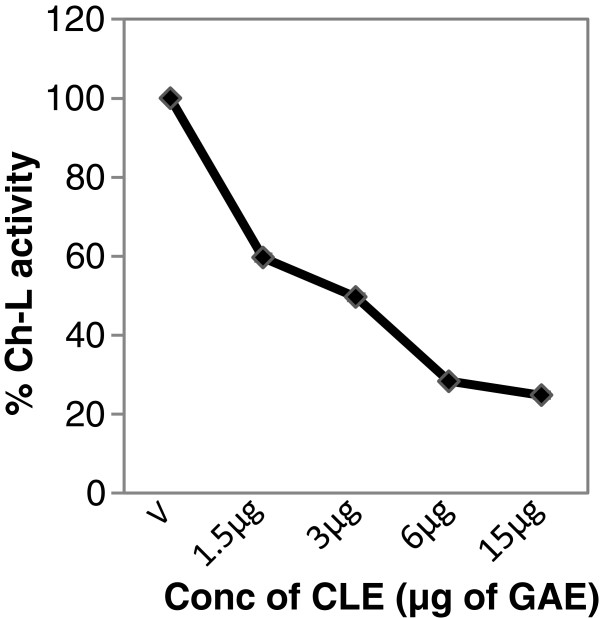
**Curry leaf extract inhibits activity of purified 20S proteasome.** Purified 20S proteasome from rabbit was incubated with 40 μM of fluorogenic peptide substrate for Ch-L activity in the presence of various concentrations of the CLE for 2 h at 37°C. The fluorescence intensity of the free AMCs was determined using fluorescence mode in a multimode reader with excitation (380 nm) and emission (460 nm).

We then tested whether or not CLE inhibited the activity of the purified 20S rabbit proteasome in a cell-free system. Indeed CLE decreased the chymotrypsin-like activity of the 20S proteasome in a dose-dependent manner and a 50% decrease (**IC**_**50**_) in activity was seen at a concentration of CLE equivalent to 3 ug of Gallic Acid (GAE)/μl of the extract (Figure 8).

### M. *koenigii* leaf extract inhibits cellular 26S proteasome activity in intact cells

**Figure 9:**
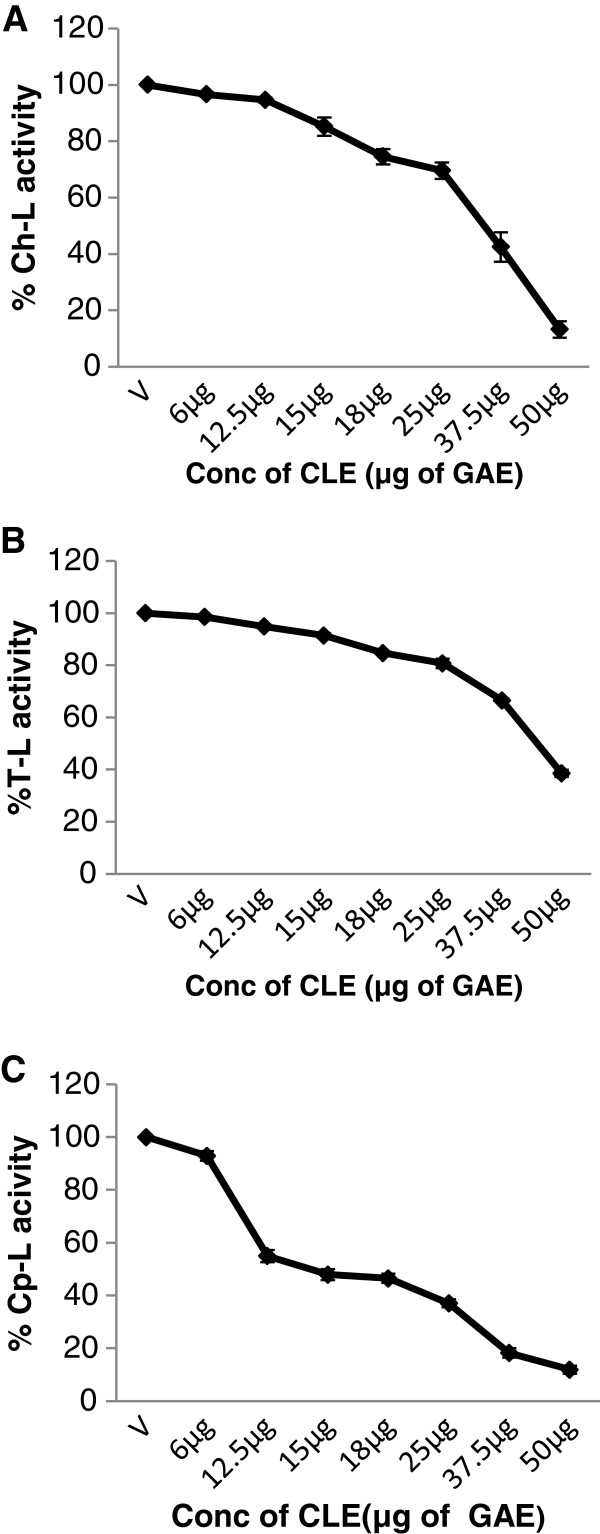
**Inhibition of the enzymatic activities of the 26S proteasome by the CLE in intact MDA-MB-231 breast cancer cell line.** Intact **MDA-MB-231** were treated for 24 h with or without the CLE followed by 2 h incubation at 37°C with the fluorogenic substrate for Ch-L or T-L or Cp-L activities respectively. The fluorescent intensity of the free AMCs was determined in a multimode reader with excitation (380 nm) and emission (460 nm). Each activity was expressed as the percentage of the control (defined as 100%). Panel **A**, **B** &**C** represents Ch-L, T-L and Cp-L activities respectively.

**Figure 10:**
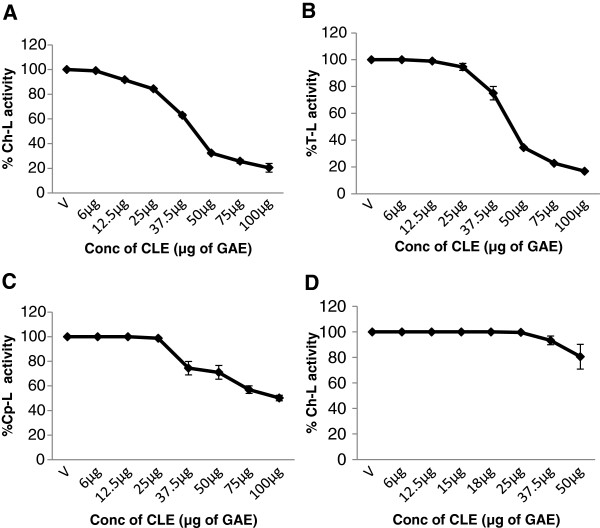
**Inhibition of the enzymatic activities of the 26S proteasome by the CLE in intact MCF-7 breast cancer cell line and WI-38 normal cell line.** Intact **MCF-7 or WI-38** cells were treated for 24 h with or without the CLE followed by 2 h incubation at 37°C with the fluorogenic substrate for either Ch-L or T-L or Cp-L activities respectively. The fluorescent intensity of the free AMCs was determined in a multimode reader with excitation (380 nm) and emission (460 nm). Each activity was expressed as the percentage of the control (defined as 100%). Panel **D** depicts results from **WI-38 cells**. Panels **A** &**D** represents Ch-L activity; panels **B** &**C** depicts T-L and Cp-L activities respectively.

Whether the CLE also inhibited the activity of the 26S proteasome in living cancer cells was assessed next in both MCF-7 and MDA-MB-231 cells. Similar to its effects on the purified 20S rabbit proteasome, CLE showed a significant (p<0.05), dose-dependent decrease in the chymotrypsin-like, trypsin-like and caspase-like activities of the 26S proteasome in intact cancer cells (Figures 9 and 10).

On the other hand, CLE did not inhibit the chymotrypsin-like activity of the 26S proteasome at any of the concentrations tested in the normal WI-38 cells (Figure 10D) indicating the specificity of the effect to cancer cells.

### M. *koenigii* leaf extract inhibits cellular 26S proteasome activity in cell extracts

**Figure 11:**
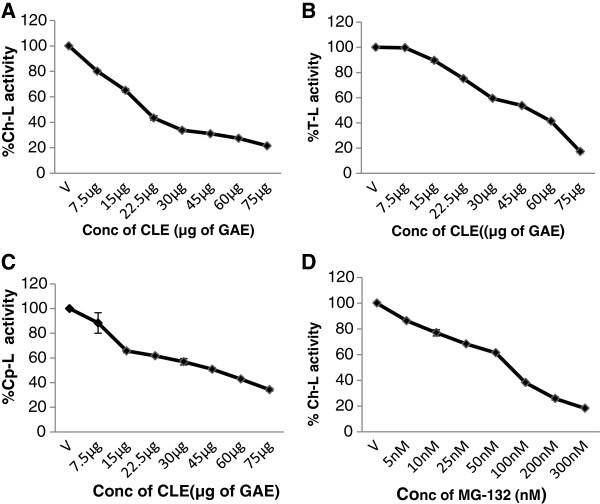
**Concentration-dependent inhibition of the enzymatic activities of the 26S proteasome by the CLE or MG-132 in MDA-MB-231 cell extracts.** Cell extracts (=50 μg protein) were incubated with 50 μM of the fluorogenic substrates specific for Ch-L, T-L and Cp-L activities in the presence of various concentrations of the CLE for 2 h at 37°C. Each activity was expressed as the percentage of the control (defined as 100%). Panel **A**, **B** &**C** depicts Ch-L, T-L and Cp-L activities respectively, whereas panel **D** depict Ch-L activity with the synthetic proteasome inhibitor MG-132.

**Figure 12:**
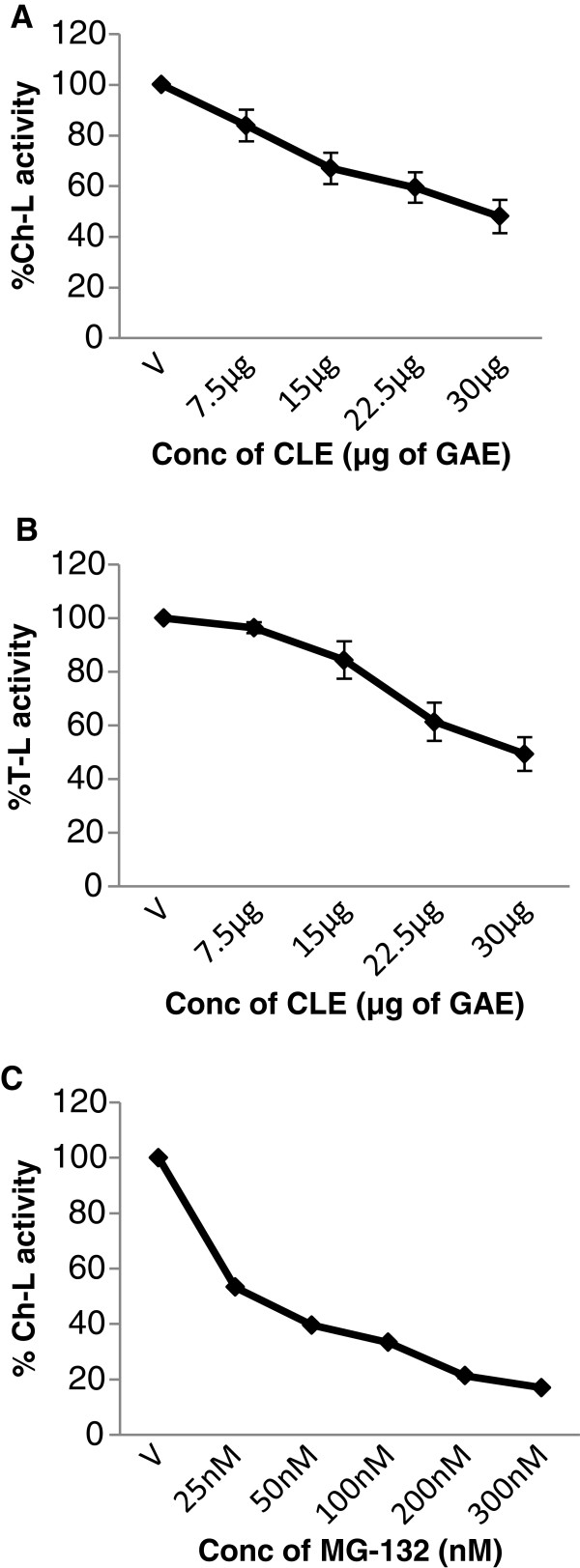
**Concentration-dependent inhibition of the enzymatic activities of the 26S proteasome by the CLE or MG-132 in MCF-7 cell extracts.** Cell extracts (=50 μg protein) were incubated with 50 μM of the fluorogenic substrates specific for Ch-L, T-L and Cp-L activities in the presence of various concentrations of the CLE for 2 h at 37°C. Each activity was expressed as the percentage of the control (defined as 100%). Panel **A** &**B** depicts Ch-L and T-L activities respectively, whereas panel **C** depicts Ch-L activity with MG-132.

Further to confirm that the CLE inhibits the 26S proteasome, cell extracts enriched in 26S proteasomes were prepared from both MCF-7 and MDA-MB-231 cells. The cell extracts were then treated with the CLE and inhibition of the three proteasomal activities was assessed. It was interesting that CLE inhibited the chymotrypsin-like, trypsin-like and caspase-like activities of the 26S proteasome in cell extracts in a dose-dependent manner in both MDA-MB-231 [IC_50_ of 22.5 μg CLE] and MCF-7 cells [IC_50_ of 30 μg CLE] (Figures 11 and 12). As a positive control MG-132, a proteasome inhibitor was also tested. It was observed that MG-132 decreased the chymotrypsin-like activity of the 26S proteasome in a dose-dependent manner in cell extracts prepared from both MDA-MB-231 and MCF-7 cells with an IC_50_ of >50 nM and 25 nM in the two cell lines respectively (Figures 11D &12C).

## Discussion

Search for new anti-cancer drugs from natural sources is one of the most important approaches for cancer prevention and treatment. In recent years, more emphasis is laid on Complementary and Alternative [CAM] forms of medicine for the treatment of various cancers, among which herbal medicine is now being explored for cancer therapy [36]. Dietary constituents have chemopreventive and chemotherapeutic potential, in addition to ameliorating the side effects associated with conventional chemotherapy. In this context, a recent approach in cancer therapy advocates the inhibition of the proteolytic activity of 26S proteasome, the multi-enzymatic protease complex in cells. Unlike normal cells, cancer cells have increased proteasomal activity which is essential for their survival and uninhibited proliferation [37-39]. Inhibition of the proteasome results in apoptosis and cancer cell death [40]. Importantly, inhibitors of the 20S proteolytic unit of the proteasome have been shown to induce apoptosis and cell cycle arrest only in neoplastic cells but not in normal cells [39-41]. Therefore, the proteasome has emerged as an attractive molecular target for cancer therapy [42]. A number of synthetic proteasome inhibitors have been described and most of them interfere with the proteolytic activity of the β subunits of the 20S proteasome. These inhibitors which bind the active site either reversibly or irreversibly include peptide aldehydes such as MG-132, non-peptide inhibitors such as lactacystin and epoxomycin and peptide boronates such as bortezomib [43].

Bortezomib/Velcade/PS-341 is the first-in-line and the only dipeptide boronate proteasome inhibitor to be approved by the FDA in 2003 for the treatment of patients with refractory multiple myeloma. Bortezomib is now being tested in a variety of hematological and solid tumors including non-Hodgkin’s lymphoma, prostate, breast and non-small-cell-lung cancer [44,45]. In recent years, synthetic polyphenols such as apigenin, epigallocatechin gallate [EGCG], quercitin and myrcetin have been reported to act as proteasome inhibitors and induce cell death in cancer cells [24].

Drug resistance limits the effectiveness of existing treatment options and is a major challenge faced in current cancer research. Interestingly, it has been shown that lactacystin and bortezomib enhance sensitivity of cancer cells that are resistant to routine chemotherapy [46,47]. Nevertheless, synthetic proteasome inhibitors are associated with some toxicity. Therefore, proteasome inhibitors from natural food sources with minimal or no toxicity can be potential anticancer agents.

In the present study, we report the anticancer potential of M. *koenigii* leaf extracts in two human breast carcinoma cell lines. In recent years, dietary polyphenols have attracted lot of attention owing to their anti-tumor activities [48,49]. One such activity is the inhibition of the proteasome in cancer cells leading to cell death. Recent work from our laboratory [21] has demonstrated that M. *koenigii* leaf extract is a rich source of polyphenols. In this study, we found that a hydro-methanolic extract of curry leaves is rich in polyphenol content. Extracts of M. *koenigii* leaves have been reported to possess various biological activities such as anti-diabetic, anti-oxidative and anti-inflammatory [8-10]. Recently, carbazole alkaloids from M. *koenigii* have shown anti-cancer activity in leukemia cells [18-20]. However, the underlying mechanism(s) are not reported yet. In the present work, we demonstrate *for the first time* that the hydro-methanolic extract of curry leaf has proteasome-inhibitory potential and induces cell death in human breast cancer cells.

We found that the methanolic extract of curry leaves significantly decreased cell viability and proliferation of both MCF-7 and MDA-MB-231 breast cancer cells in a dose-dependent manner. This was further supported by the significant reduction in the number of colonies in CLE treated cells compared to vehicle treated cells. Our cell viability and colony formation data shows that CLE altered the growth kinetics of both MCF-7 and MDA-MB-231 cells. Therefore, curry leaves appear to be a promising drug candidate for restricting the growth of breast cancer cells.

In order to assess the stage at which the cell growth was arrested by CLE, we performed cell cycle experiments and observed that there was a clear arrest of cells in the synthetic or S phase. In contrast to its effect on the breast carcinoma cell lines, CLE interestingly, had no effect on the different phases of the cell cycle in the normal fibroblast cell line. Anti-cancer drugs can result either in programmed cell death/apoptosis or necrosis. In order to identify the probable cell death pathway involved, we used Annexin V binding to test if the cell death occurred through apoptosis or necrosis. Indeed it was found that in both the breast cancer cell lines CLE induced apoptotic cell death.

We tested next, whether the anti-cancerous effect of the CLE was due its potential of inhibiting the proteolytic activity of the protein degrading machine present in eukaryotic cells – the 26S proteasome, which is now considered to be a novel approach for cancer therapy. We observed that CLE inhibited the purified 20S proteasome enzyme. Furthermore, it significantly inhibited all the three enzymatic activities associated with the 26S proteasome in living cells in a dose-dependent manner. To further confirm the findings in live cells, cell extracts were prepared from both breast cancer cells and tested for the potential of the CLE in inhibiting the cellular proteasome. Similar to our findings in intact cells we found that CLE inhibited the 26S proteasome in cell extracts also in a dose-dependent manner. To test whether or not the inhibitory effect of the CLE on the proteasome activity was specific to cancer cells, we tested its effects in WI-38, a normal lung fibroblast cell line. Interestingly, CLE had no effect on the chymotrypsin–like activity of the 26S proteasome in live WI-38 cells. Hence, data from our proteasome-inhibition experiments suggests that the CLE could inhibit the cellular proteasome leading to cell death in cancer cells but not normal cells. Our data is in accordance with earlier reports that proteasome inhibitors selectively inhibited proteasome activity only in neoplastic cells [39,50,51].

## Conclusion

Our results indicate that the hydro-methanolic extract of curry leaves is a good source of active compound(s) that can potentially inhibit the 26S proteasome specifically in cancer cells. The inhibition of the proteasome in cancer cells appears to be one of the important biological activities in M. *koenigii* leaves that can be exploited for cancer treatment. Hence, isolation and characterization of active component(s) from methanolic extracts of curry leaves could lead to the discovery of novel anticancer agents.

## Abbreviations

(MTT): 3-(4,5-dimethylthiazol-2-yl)-2,5-di-phenyltetrazolium bromide; (GAE): Gallic Acid Equivalents; (CLE): Curry leaf extract; (AMC): 7-amino-4-methylcoumarin; (DMSO): Dimethyl sulphoxide; (EGCG): Epigallocatechin gallate; (DTT): Dithiothretol; (FITC): Fluorescein isothiocyanate; (PBS): Phosphate buffered saline.

## Competing interests

The authors declare that they have no competing interests.

## Authors’ contributions

AYI designed the study and wrote the manuscript. BN carried out most of the experiments. RA assisted with the cell cycle and Annexin-V experiments and data analysis. AC helped with cytotoxicity assays. BKN has done the statistical analysis of the data. RM has critically read and revised the manuscript for intellectual content. All authors read and approved the final manuscript.

## Pre-publication history

The pre-publication history for this paper can be accessed here:

http://www.biomedcentral.com/1472-6882/13/7/prepub

## References

[B1] Parkin DM Bray F Ferlay J Pisani P Estimating the world cancer burden: Globocan Int J Cancer 2000 94 153 156 11668491 10.1002/ijc.1440

[B2] Pruthi JS Spices and condiments 1976 New Delhi: National Book Trust India

[B3] Chevallier A The encyclopedia of medicinal plants 1996 London: Dorlon Kindersley Publisher

[B4] Sivarajan VV Balachandran I Ayurvedic drugs and their plant sources 1994 New Delhi: Oxford & IBH Publishing

[B5] Muthumani P Venkatraman S Ramseshu KV Meera R Devi P Kameswari B Pharmacological studies of anticancer, anti inflammatory activities of Murraya koenigii (Linn) Spreng in experimental animals J Pharm Sci Res 2009 1 137 141

[B6] Nayak A Banerji J Banerji A Mandal S Review on chemistry and pharmacology of Murraya koenigii Spreng (Rutaceae) J Chem Pharm Res 2010 2 286 299

[B7] Kesari AN Kesari S Singh SK Gupta RK Watal G Studies on the glycemic and lipidemic effect of Murraya koenigii in experimental animals J Ethnopharmacol 2007 112 2 305 311 10.1016/j.jep.2007.03.023 17467937

[B8] Arulselvan P Senthilkumar GP Sathish Kumar D Subramanian S Anti-diabetic effect of Murraya koenigii leaves on streptozotocin induced diabetic rats Pharmazie 2006 61 10 874 877 17069429

[B9] Lawal HA Atiku MK Khelpai DG Hypoglycaemic and hypolipidemic effect of aqueous leaf extract of Murraya koenigii in normal and alloxan-diabetic rats Niger J Physiol Sci 2008 23 1–2 37 40 19434212 10.4314/njps.v23i1-2.54919

[B10] Gupta S Prakash J Studies on Indian green leafy vegetables for their antioxidant activity Plant Foods Hum Nutr 2009 64 1 39 45 10.1007/s11130-008-0096-6 18985454

[B11] Tachibana Y Kikuzaki H Lajis NH Nakatani N Antioxidative activity of carbazoles from Murraya koenigii leaves J Agric Food Chem 2001 49 11 5589 5594 10.1021/jf010621r 11714364

[B12] Khan BA Abraham A Leelamma S Anti-oxidant effects of curry leaf, Murraya koenigii and mustard seeds, Brassica juncea in rats fed with high fat diet Indian J Exp Biol 1997 35 2 148 150 9315222

[B13] Adebajo AC Ayoola OF Iwalewa EO Akindahunsi AA Omisore NO Adewunmi CO Adenowo TK Anti-trichomonal, biochemical and toxicological activities of methanolic extract and some carbazole alkaloids isolated from the leaves of Murraya koenigii growing in Nigeria Phytomedicine 2006 13 4 246 254 10.1016/j.phymed.2004.12.002 16492527

[B14] Mandal S Nayak A Kar M Banerjee SK Das A Upadhyay SN Singh RK Banerji A Banerji J Antidiarrheal activity of carbazole alkaloids from Murraya koenigii Spreng (Rutaceae) seeds Fitoterapia 2010 81 1 72 74 10.1016/j.fitote.2009.08.016 19695314

[B15] Xie JT Chang WT Wang CZ Mehendale SR Li J Ambihaipahar R Ambihaipahar U Fong HH Yuan CS Curry leaf (Murraya koenigii Spreng.) reduces blood cholesterol and glucose levels in ob/ob mice Am J Chin Med 2006 34 2 279 284 10.1142/S0192415X06003825 16552838

[B16] Gupta S George M Singhal M Sharma GN Garg V Leaves extract of murraya koenigii linn for anti-inflammatory and analgesic activity in animal models J Adv Pharm Technol Res 2010 1 1 68 77 22247833 PMC3255384

[B17] Shah AS Wakade AS Juvekar AR Immunomodulatory activity of methanolic extract of Murraya koenigii (L) Spreng. Leaves Indian J Exp Biol 2008 46 7 505 509 18807753

[B18] Ito C Itoigawa M Nakao K Murata T Tsuboi M Kaneda N Furukawa H Induction of apoptosis by carbazole alkaloids isolated from Murraya koenigii Phytomedicine 2006 13 5 359 365 10.1016/j.phymed.2005.03.010 16635744

[B19] Roy MK Thalang VN Trakoontivakorn G Nakahara K Mechanism of mahanine-induced apoptosis in human leukemia cells (HL-60) Biochem Pharmacol 2004 67 1 41 51 10.1016/j.bcp.2003.07.021 14667927

[B20] Bhattacharya K Samanta SK Tripathi R Mallick A Chandra S Pal BC Shaha C Mandal C Apoptotic effects of mahanine on human leukemic cells are mediated through crosstalk between Apo-1/Fas signaling and the Bid protein and via mitochondrial pathways Biochem Pharmacol 2010 79 3 361 372 10.1016/j.bcp.2009.09.007 19751707

[B21] Ayesha I Bindu N Shulagna S Chandana M Mehrajuddin B Raghunath M Proteasome inhibitory potential of commonly consumed dietary ingredients Int J Food Nutr Sci 2012 1 4 27 31

[B22] Scalbert A Manach C Morand C Rémésy C Jiménez L Dietary polyphenols and the prevention of diseases Crit Rev Food Sci Nutr 2005 45 4 287 306 Review 10.1080/1040869059096 16047496

[B23] Scalbert A Johnson IT Saltmarsh M Polyphenols: antioxidants and beyond Am J Clin Nutr 2005 81 Suppl 1 215S 217S Review 15640483 10.1093/ajcn/81.1.215S

[B24] Chen D Daniel KG Chen MS Kuhn DJ Landis-Piwowar KR Dou QP Dietary flavonoids as proteasome inhibitors and apoptosis inducers in human leukemia cells Biochem Pharmacol 2005 69 10 1421 1432 10.1016/j.bcp.2005.02.022 15857606

[B25] Pettinari A Amici M Cuccioloni M Angeletti M Fioretti E Eleuteri AM Effect of polyphenolic compounds on the proteolytic activities of constitutive and immuno-proteasomes Antioxid Redox Signal 2006 8 1–2 121 129 16487045 10.1089/ars.2006.8.121

[B26] Hershko A Ciechanover A The ubiquitin system Annu Rev Biochem 1998 67 425 479 Review 10.1146/annurev.biochem.67.1.425 9759494

[B27] Groll M Ditzel L Lowe J Stock D Bochtler M Bartunik HD Huber R Structure of 20S proteasome from yeast at 2.4 A resolution Nature 1997 386 463 471 10.1038/386463a0 9087403

[B28] Rivett AJ The multicatalytic proteinase. Multiple proteolytic activities J Biol Chem 1989 264 21 12215 12219 2745438

[B29] Jung T Bader N Grune T Oxidized proteins: intracellular distribution and recognition by the proteasome Arch Biochem Biophys 2007 462 2 231 237 Review 10.1016/j.abb.2007.01.030 17362872

[B30] Tambyrajah WS Bowler LD Medina-Palazon C Sinclair AJ Cell cycle-dependent caspase-like activity that cleaves p27(KIP1) is the beta(1) subunit of the 20S proteasome Arch Biochem Biophys 2007 466 2 186 193 10.1016/j.abb.2007.07.019 17854759

[B31] Chen WJ Lin JK Induction of G1 arrest and apoptosis in human jurkat T cells by pentagalloylglucose through inhibiting proteasome activity and elevating p27Kip1, p21Cip1/WAF1, and Bax proteins J Biol Chem 2004 279 14 13496 13505 10.1074/jbc.M212390200 14726525

[B32] Hilt W Wolf DH Proteasomes: The World of Regulatory Proteolysis 2000 Georgetown, Texas: Landes Bioscience

[B33] Goldberg AL Functions of the proteasome: the lysis at the end of the tunnel Science 1995 268 5210 522 523 Review 10.1126/science.7725095 7725095

[B34] Hochstrasser M Ubiquitin, proteasomes, and the regulation of intracellular protein degradation Curr Opin Cell Biol 1995 7 2 215 223 Review 10.1016/0955-0674(95)80031-X 7612274

[B35] Singleton VL Orthofer R Lamuela-Raventos RM Analysis of total phenols and other oxidation substrates and antioxidants by means of Folin-Ciocalteu Reagent Methods Enzymol 1999 299 152 178

[B36] Adams M Jewell AP The use of complementary and alternative medicine by cancer patients Int Semin Surg Oncol 2007 4 10 10.1186/1477-7800-4-10 17470282 PMC1872026

[B37] Wada M Kosaka M Saito S Sano T Tanaka K Ichihura A Serum concentrations and localization in tumor cells of proteasomes in patients with hematologic malignancy and their pathophysiologic significance J Lab Clin Med 1993 121 215 223 8433038

[B38] Li B Dou QP Bax degradation by the ubiquitin/proteasome-dependent pathway: involvement in tumor survival and progression Proc Natl Acad Sci USA 2000 97 8 3850 3855 10.1073/pnas.070047997 10725400 PMC18105

[B39] An B Goldfarb RH Siman R Dou QP Novel dipeptidyl proteasome inhibitors overcome Bcl-2 protective function and selectively accumulate the cyclin-dependent kinase inhibitor p27 and induce apoptosis in transformed, but not normal, human fibroblasts Cell Death Differ 1998 5 12 1062 1075 10.1038/sj.cdd.4400436 9894613

[B40] Dou QP Li B Proteasome inhibitors as potential novel anticancer agents Drug Resist Update 1999 2 4 215 223 10.1054/drup.1999.0095 11504494

[B41] Kazi A Urbizu DA Kuhn DJ Acebo AL Jackson ER Greenfelder GP Kumar NB Dou QP A natural musaceas plant extract inhibits proteasome activity and induces apoptosis selectively in human tumor and transformed, but not normal and non-transformed, cells Int J Mol Med 2003 12 6 879 887 14612961

[B42] Landis-Piwowar KR Milacic V Chen D Yang H Zhao Y Chan TH Yan B Dou QP The proteasome as a potential target for novel anticancer drugs and chemosensitizers Drug Resist Update 2006 9 6 263 273 Review 10.1016/j.drup.2006.11.001 17197231

[B43] Adams J The proteasome: structure, function, and role in the cell Cancer Treat Rev 2003 29 Suppl 1 3 9 Review 12738238 10.1016/s0305-7372(03)00081-1

[B44] Richardson PG Hideshima T Anderson KC Bortezomib (PS-341): a novel, first-in-class proteasome inhibitor for the treatment of multiple myeloma and other cancers Cancer Control 2003 10 5 361 369 Review 14581890 10.1177/107327480301000502

[B45] Richardson PG Anderson KC Bortezomib: a novel therapy approved for multiple myeloma Clin Adv Hematol Oncol 2003 1 10 596 600 Review 16258456

[B46] Delic J Masdehors P Omura S Cosset JM Dumont J Binet JL Magdelénat H The proteasome inhibitor lactacystin induces apoptosis and sensitizes chemo- and radio resistant human chronic lymphocytic leukemia lymphocytes to TNF-alpha-initiated apoptosis Br J Cancer 1998 77 7 1103 1107 10.1038/bjc.1998.183 9569046 PMC2150120

[B47] Ma MH Yang HH Parker K Manyak S Friedman JM Altamirano C Wu ZQ Borad MJ Frantzen M Roussos E Neeser J Mikail A Adams J Sjak-Shie N Vescio RA Berenson JR The proteasome inhibitor PS-341 markedly enhances sensitivity of multiple myeloma tumor cells to chemotherapeutic agents Clin Cancer Res 2003 9 3 1136 1144 12631619

[B48] Kandaswami C Lee LT Lee PP Hwang JJ Ke FC Huang YT Lee MT The antitumor activities of flavonoids In Vivo 2005 19 895 909 16097445

[B49] Thomasset SC Berry DP Garcea G Marczylo T Steward WP Gescher J Dietary polyphenolic phytochemicals - promising cancer chemopreventive agents in humans? A review of their clinical properties Int J Cancer 2007 120 451 458 10.1002/ijc.22419 17131309

[B50] Nam S Smith DM Dou QP Ester bond-containing tea polyphenols potently inhibit proteasome activity in vitro and in vivo J Biol Chem 2001 276 16 13322 13330 10.1074/jbc.M004209200 11278274

[B51] Kuhn DJ Lam WH Kazi A Daniel KG Sung S Chow LMC Chan TH Dou QP Synthetic peracetate tea polyphenols as potent proteasome inhibitors and apoptosis inducers in human cancer cells Front Bio 2005 10 1010 1023 10.2741/1595 15769601

